# Quality of Life After Orthopedic Procedures at Buraydah Central Hospital and King Fahad Specialist Hospital in Qassim Region, Saudi Arabia

**DOI:** 10.7759/cureus.30835

**Published:** 2022-10-29

**Authors:** Muslet Alharbi, Saleh F Aldubayyan, Thamer K Alharbi, Ali K Alshaya, Faisal A Almesned, Azzam H Alsughayyir, Abdulaziz A Alkhalaf, Alzubar A Wali, Yousif H Alamri

**Affiliations:** 1 Family and Community Medicine, College of Medicine, Qassim University, Buraydah, SAU; 2 Orthopedics, College of Medicine, Qassim University, Buraydah, SAU; 3 Orthopedics, Buraydah Central Hospital, Buraydah, SAU; 4 General Practice, King Fahad Specialist Hospital, Buraydah, SAU; 5 Orthopedics, King Fahad Specialist Hospital, Buraydah, SAU

**Keywords:** mental health, orthopedic patients, surgery, sf-36, quality of life

## Abstract

Aim

Patients' perspectives on their condition and treatment, their sense of need for healthcare, and their preferences for care and outcomes are all addressed by quality of life metrics. Therefore, it is important to all health professionals and patients involved in orthopedic surgery. This study aimed to evaluate the quality of life after orthopedic procedures and how its results could potentially be used for future improvement.

Methods

This is a cross-sectional study conducted among patients who underwent orthopedic procedures at Buraydah Central Hospital (BCH) and King Fahad Specialist Hospital (KFSH). A self-administered questionnaire was distributed among the patients using a paper questionnaire. The questionnaire was composed of socio-demographic data (e.g. age, gender, education, etc.) and the 36-Item Short Form Survey (SF-36) to measure patients’ quality of life.

Results

In this study, 215 patients were able to complete the survey (male 82.3% vs female 17.7%). The most common age group was 18 to 30 years old (30.2%) and the most common surgery performed on patients was thigh surgery (19.5%) and hand surgery (13%). The overall mean physical health score after the surgery was 51.1 (SD 11.8) higher than the mental health score (mean 47.7; SD 11.2). Poor quality of life was significantly more common among patients with chronic disease while poor physical functioning and general health subdomains were more associated among patients who underwent hand surgery.

Conclusion

Patients suffering from chronic diseases tend to exhibit a low quality of life as compared to other patients. The quality of life after the surgery is an important indicator of patient satisfaction which may have a direct impact on the future outlook of a patient. More research is needed to determine the overall quality of life in patients who underwent a surgical procedure in our region.

## Introduction

Musculoskeletal problems are the most common cause of chronic pain and disability among people across the globe [1] which is a serious health issue with serious repercussions for society and the economy all over the world. According to the 2017 Global Burden Disease report, musculoskeletal problems were the leading cause of disability worldwide [2]. Musculoskeletal problems affect about a third of the world's population. As a result, they are the most significant cause of chronic disability, which frequently results in an inability to work, absenteeism, decreased job satisfaction, increased incidence of work-related injuries [3-6], and an impact on quality of life [7]. Particularly, health-related quality of life which represents mainly the patient’s functional ability, and physical, psychological, and social well-being is crucial for orthopedic surgical management [8].

In many jurisdictions, it is reported that surgical interventions for surgical correction of hallux valgus, primarily single-level transforaminal lumbar interbody fusion, total knee replacement, total hip replacement [9] arthroscopic rotator cuff repair, and treatment of fractured femur significantly improved health-related quality of life [10]. Therefore, measuring of patient’s quality of life after surgical intervention is a useful tool to assess the outcome of surgical management [11]. According to Djukanovic et al., the need for useful indicators to measure recovery, health, and quality of life is growing in healthcare settings [12].

A high level of health-related quality of life is a major goal of surgical interventions, so it should be considered when choosing a treatment. Unfortunately, very little is known about how surgical interventions affect patients' quality of life in Saudi Arabia. Therefore, this study aims to assess the health-related quality of life of patients who have undergone orthopedic surgeries. Consequently, the following research objectives were explored (1) to evaluate the quality of life regarding mobility, self-care, usual activities, pain/discomfort, and anxiety/depression after orthopedic procedures, (2) to identify factors that help to improve the quality of life after orthopedic procedures, (3) to explore the impact of modern lifestyle habits on quality of life after orthopedic procedures.

## Materials and methods

Study design

This cross-sectional study of orthopedic surgery patients was conducted at Buraydah Central Hospital (BCH) and King Fahad Specialist Hospital (KFSH) in the Qassim region between November 2020 and November 2021. KFSH is the region’s largest hospital, with more than 430 beds, and is also the region’s only tertiary care center. The hospital is located in Buraydah, the capital of the Qassim region, which is one of 13 administrative regions and is located in the center of Saudi Arabia, with a population of approximately 1.4 million. Approximately half of the population is aged less than 24 years, and one-fifth of the population has a university education or higher; the average family income in this region ranked third compared to other regions.

Study setting

This study was conducted in the Qassim region of Saudi Arabia at Buraydah Central Hospital and King Fahad Specialist Hospital.

Sample and sample size

The sample size was computed using OpenEpi statistical software (www.OpenEpi.com), assuming adequate power (80%) and an alpha of 0.05. The minimum required sample size was 194 patients. In total, we recruited 215 patients who underwent orthopedic surgery.

Sampling technique

Patients who underwent orthopedic surgery were selected following admission to BCH and KFSH and during scheduled visits for assessment. Patients with cancer or major psychotic disorders were excluded from the study.

Data collection methods

To assess HRQoL, we used the RAND 36-Item Health Survey 1.0 Questionnaire in Arabic (the 36-item Short Form (SF-36) Health Survey) which is a validated, self-reported questionnaire. The instrument comprises 36 items with two composite measures of physical and mental components that encompass the following eight domains: physical functioning (PF), role limitations due to physical functioning (RP), bodily pain (BP), general health perceptions (GH), vitality (VT), social functioning (SF), role limitations due to emotional health (RE), and general mental health (MH). Each item is scored from 0 to 100, representing the worst to the best quality of life. The physical component summary (PCS) score is calculated as the aggregate of four domains: PF (10 items), RP (four items), BP (two items), and GH (five items). The mental component summary (MCS) score is calculated as the aggregate of RE (three items), VT (four items), SF (two items), and MH (five items). The study’s purpose and objectives were explained to eligible patients, and those who consented to participate were interviewed face-to-face by trained interviewers using the standard validated SF-36 questionnaire for HRQoL assessment. The study was approved by the Regional Qassim Ethics Committee and conformed to the ethics guidelines of the Declaration of Helsinki. Written informed consent was obtained from all participants in the study.

Ethical considerations

The following ethical guidelines were established: (1) informed consent will be clear and indicates the purpose of the study and the right of the participant to withdraw at any time without any obligation towards the study team, (2) the participant’s anonymity will be assured by assigning each participant with a code number for the purpose of analysis only, (3) no incentives or rewards will be given to participants, and (4) institutional review board approval will be obtained for this study prior to execution.

Statistical analysis

Descriptive statistics were summarized as numbers, percentages, mean, standard deviation, and median (min-max). The differences in the score of the domains and subdomains of quality of life in relation to the selected socio-demographic characteristics and the name of surgery were calculated using the Mann-Whitney U-test as well as the Kruskal-Wallis H-test. The normality test was performed using the Shapiro-Wilk test and Kolmogorov-Smirnov test. The domains and subdomains of quality of life followed the abnormal distribution. Thus, non-parametric tests were applied. A p-value of 0.05 was considered statistically significant. The data were analyzed using Statistical Packages for Social Sciences software, v.26 (IBM Corp., Armonk, NY).

## Results

A total of 215 patients who underwent orthopedic procedures were recruited. Table 1 presents the socio-demographic characteristics of the patients. Around 30.2% were aged between 18 to 30 years old with nearly all (82.3%) being males. Patients who finished secondary school were 39.1%. Most of the patients had no associated chronic disease (86%); 82.3% of the patients underwent surgical intervention in the last two months or less, and 57.2% indicated that their surgery was decided in the OPD.

**Table 1:** Socio-demographic characteristics of the patients who underwent orthopedic procedures (N = 215)

Study variables	N (%)
Age group	
<18 years	33 (15.3%)
18 – 30 years	65 (30.2%)
31 – 40 years	45 (20.9%)
41 – 50 years	24 (11.2%)
51 – 60 years	18 (08.4%)
>60 years	30 (14.0%)
Gender	
Male	177 (82.3%)
Female	38 (17.7%)
Educational level	
Uneducated	13 (06.0%)
Primary school	44 (20.5%)
Middle school	47 (21.9%)
Secondary school	84 (39.1%)
Bachelor’s degree	26 (12.1%)
Master’s degree	01 (12.1%)
Associated chronic disease	
DM	11 (05.1%)
HTN	11 (05.1%)
Asthma	01 (0.50%)
Thyroid disease	02 (0.90%)
DM & HTN	05 (02.3%)
None	185 (86.0%)
Duration since surgery	
≤2 months	177 (82.3%)
>2 months – 4 months	25 (11.6%)
>4 months – 6 months	05 (02.3%)
>6 months	08 (03.7%)
Location where surgery has been decided	
ER	92 (42.8%)
OPD	123 (57.2%)

**Figure 1:**
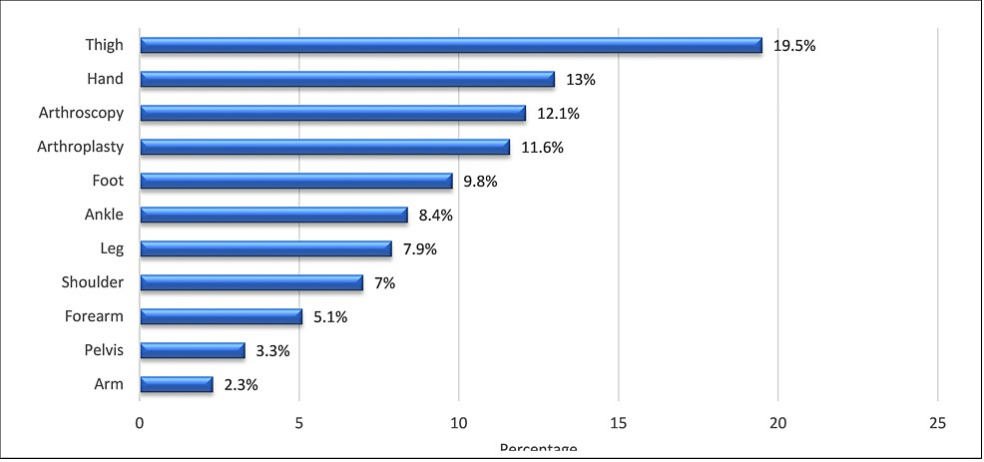
The specific type of surgery performed on patients

Figure 1 depicted the specific type of surgery done among patients. It can be observed that the most common surgical procedure performed on patients was thigh surgery (19.5%), followed by hand surgery (13%) and arthroscopy (12.1%).

Furthermore, in the physical health subdomains, the physical functioning subdomain showed the highest mean rating (mean 55.8), followed by body pain (mean 52.6). The overall mean score for physical health was 51.1 (SD 11.8). For mental health subdomains, social functioning was the highest (mean 53.3) followed by role limitations due to emotional problems (mean 52.2). The overall mean score for the mental health domain was 47.7 (SD 11.2) Table 2 shows details of the assessment of the quality of life according to the SF-36 Short-Form Survey. 

**Table 2:** Assessment of quality of life according to SF-36 short form (n = 215)

QoL domains	Mean	SD	Median	Min	Max
Physical functioning	55.8	27.3	55.0	0.00	100
Role limitations due to physical health	49.3	37.2	50.0	0.00	100
Body pain	52.6	15.3	52.5	0.00	100
General health	46.7	10.6	50.0	15.0	75.0
Physical health score	51.1	11.8	51.2	21.9	82.5
Role limitations due to emotional problems	52.2	39.9	66.7	0.00	100
Energy/fatigue	41.7	13.2	40.0	5.00	100
Emotional well-being	43.6	16.3	48.0	0.00	100
Social functioning	53.3	16.8	50.0	0.00	100
Mental Health score	47.7	11.2	48.3	19.4	78.1

Additionally, the findings indicated a higher emotional well-being score (p=0.007) and total mental health score (p=0.005) were more associated with the younger age group (p=0.007). However, the scores of other QoL domains did not vary significantly between younger (≤35 years) and older (>35 years) age groups (p>0.05). Table 3 shows the differences in quality of life according to age.

**Table 3:** Differences in quality of life according to age (n = 215) § P-value has been calculated using Mann-Whitney U-test. ** Significant at p<0.05 level.

QoL domains	≤35 years Mean ± SD	>35 years Mean ± SD	P-value ^§^
Physical functioning	54.2 ± 27.7	57.8 ± 26.7	0.406
Role limitations due to physical health	51.9 ± 37.7	46.2 ± 36.5	0.268
Pain	53.8 ± 15.3	51.2 ± 15.3	0.266
General health	47.6 ± 11.1	45.6 ± 9.85	0.082
Physical health score	51.9 ± 12.0	50.2 ± 11.5	0.220
Role limitations due to emotional problems	55.8 ± 40.1	47.9 ± 39.5	0.141
Energy/fatigue	41.8 ± 11.7	41.4 ± 14.7	0.857
Emotional well-being	46.4 ± 14.2	40.3 ± 17.9	0.007 **
Social functioning	54.4 ± 17.2	52.0 ± 16.4	0.662
Mental health score	49.6 ± 10.8	45.4 ± 11.3	0.005 **

Moreover, the findings indicated a higher emotional well-being score was more associated with being male (p=0.039), but the scores of other QoL domains including its subdomains did not reach statistical significance (p>0.05). Table 4 shows differences in quality of life according to gender.

**Table 4:** Differences in quality of life according to gender (n = 215) § P-value has been calculated using Mann-Whitney U-test. ** Significant at p<0.05 level.

QoL domains	Male Mean ± SD	Female Mean ± SD	P-value ^§^
Physical functioning	56.2 ± 26.6	54.2 ± 30.7	0.725
Role limitations due to physical health	50.9 ± 37.2	41.4 ± 37.2	0.153
Pain	51.8 ± 13.7	56.3 ± 21.3	0.589
General health	47.0 ± 10.4	45.3 ± 11.6	0.409
Physical health score	51.5 ± 12.0	49.3 ± 10.6	0.292
Role limitations due to emotional problems	41.4 ± 36.9	52.4 ± 39.0	0.983
Energy/fatigue	41.8 ± 12.9	41.2 ± 14.2	0.818
Emotional well-being	44.9 ± 15.5	37.6 ± 18.4	0.039 **
Social functioning	51.9 ± 15.5	59.9 ± 20.9	0.060
Mental health score	47.7 ± 11.0	47.6 ± 12.1	0.612

Regarding the physical health domains, lower role-physical (p=0.027), body pain (p=0.020), and total physical health (p=0.021) scores were more associated with having a chronic disease. For the mental health domain, higher energy/fatigue was more associated with having a chronic disease (p=0.006) whereas a lower social functioning score was more associated with having a chronic disease (p=0.034). Other QoL domains were not observed as relevant factors of having a chronic disease (p>0.05) Table 5 shows the differences in quality of life between patients with and without chronic disease.

**Table 5:** Differences in quality of life in patients with chronic disease compared to patients with no chronic disease (n = 215) § P-value has been calculated using Mann-Whitney U-test. ** Significant at p<0.05 level.

QoL domains	With chronic disease mean ± SD	Without chronic disease mean ± SD	P-value ^§^
Physical functioning	57.7 ± 25.6	55.5 ± 27.6	0.646
Role limitations due to physical health	35.0 ± 31.9	51.6 ± 37.6	0.024 **
Pain	45.9 ± 17.1	53.7 ± 14.8	0.020 **
General health	48.5 ± 10.8	46.4 ± 10.6	0.372
Physical health score	46.8 ± 9.76	51.8 ± 11.9	0.021 **
Role limitations due to emotional problems	46.7 ± 39.7	53.2 ± 39.9	0.429
Energy/fatigue	48.8 ± 14.7	40.5 ± 12.5	0.006 **
Emotional well-being	42.3 ± 19.8	43.8 ± 15.7	0.459
Social functioning	47.5 ± 14.1	54.3 ± 17.1	0.034 **
Mental health score	46.3 ± 11.9	47.9 ± 11.1	0.335

A statistical test revealed that all QoL domains and subdomains did not show significant differences between ER and OPD as a location where surgery has been decided (all p>0.05). Table 6 shows the differences in quality of life according to the location where surgery has been decided. 

**Table 6:** Differences in quality of life according to the location where surgery has been decided (n = 215) § P-value has been calculated using Mann-Whitney U-test.

QoL domains	ER Mean ± SD	OPD Mean ± SD	P-value ^§^
Physical functioning	53.7 ± 27.4	57.4 ± 27.2	0.391
Role limitations due to physical health	48.1 ± 35.6	50.2 ± 38.5	0.701
Pain	50.9 ± 14.8	53.9 ± 15.6	0.540
General health	47.9 ± 11.3	45.8 ± 9.96	0.086
Physical health score	50.2 ± 11.9	51.8 ± 11.7	0.303
Role limitations due to emotional problems	56.9 ± 37.5	48.8 ± 41.4	0.136
Energy/fatigue	41.7 ± 15.2	41.6 ± 11.5	0.812
Emotional well-being	44.9 ± 16.2	42.7 ± 16.4	0.292
Social functioning	51.2 ± 15.3	54.9 ± 17.8	0.143
Mental health score	48.7 ± 11.3	46.9 ± 11.1	0.349

The results also indicated a higher physical functioning score was more associated with ankle surgery but not with hand surgery (p<0.001). Also, hand surgery was more associated with a lower score in general health but a higher score in arm surgery (p=0.003). In addition, a higher role-emotional score was more associated with ankle surgery, but it was less associated with arthroplasty procedures (p=0.037). On the other hand, the differences in other HRQOL domains and subdomains did not vary significantly in each of the surgical procedures being performed among the subject patients (p>0.05). Table 7 shows the differences in the mean score of quality of life according to the type of surgery.

**Table 7:** Differences in the mean score of quality of life according to the type of surgery (n = 215) Anthrop – Anthroplasty; Anthros – Arthroscopy. § P-value has been calculated using the Kruskal-Wallis test. ** Significant at p<0.05 level

QoL domains	Leg	Arthrop	Ankle	Arthros	Thigh	Arm	Foot	Forearm	Hand	Shoulder	Pelvis	P-value ^§^
Physical functioning	65.0	63.8	71.9	54.2	66.3	54.0	55.0	46.4	33.2	35.3	60.0	<0.001 **
Role-physical health	45.6	44.0	55.6	44.2	45.8	55.0	50.0	56.8	60.7	48.3	39.3	0.847
Pain	52.6	51.3	52.5	52.9	48.6	57.0	54.0	50.0	57.7	54.3	53.2	0.786
General health	52.9	45.4	51.4	43.8	47.9	56.0	42.6	48.2	42.1	47.7	47.1	0.003 **
Physical health score	54.0	51.1	57.8	48.8	52.2	55.5	50.4	50.3	48.4	46.4	49.9	0.211
Role-emotional	37.3	33.3	74.1	48.7	48.4	66.7	50.8	60.6	65.5	57.8	52.4	0.037 **
Energy/fatigue	38.8	44.0	41.1	45.0	41.5	37.0	43.3	48.2	37.1	37.3	45.0	0.298
Emotional well-being	45.2	41.9	46.4	42.8	47.1	43.2	42.5	41.8	41.6	39.7	44.0	0.753
Social functioning	54.4	55.0	47.2	54.3	49.1	50.0	55.4	55.7	57.6	55.8	51.8	0.728
Mental health score	43.9	43.6	52.2	47.7	46.6	49.2	47.9	51.6	50.4	47.7	48.3	0.174

## Discussion

This study investigated the quality of life of patients who underwent surgical intervention at BCH and KFSH in the Qassim Region, Saudi Arabia. Using the SF-36 questionnaire, we found that the mean physical component score postoperatively (mean: 51.1) was higher than the mean mental component score (mean: 47.7). Regarding physical component domains, physical functioning showed the highest mean (mean score: 52.6) while general health was the lowest (mean score: 46.7%). In mental component domains, social functioning revealed the highest (mean score: 53.3) and energy/fatigue revealed the lowest (mean score: 41.7). Contradicting these findings, Bahardoust et al. [13] found a significantly lower mean physical component score in the patient group but not with the mental component score. In the United States [9], after 12 months of follow-up, patients showed a significant improvement in physical HRQoL and mental HRQoL which was maintained after the last follow-up; this has been mirrored across published studies where the patients' QoL improved significantly after surgical procedures [11,14-17].

Furthermore, the overall mental health score, as well as the emotional well-being score of the younger age group (≤35 years), was significantly better when compared to the older age group (>35 years) postoperatively. This is consistent with Sun et al. [18], who documented that age was positively associated with mental health HRQoL and had shown great improvement in overall HRQoL one year after limb-salvage or ablative surgery. However, in a study by Lin et al. [15], they found that patients who were aged less than 65 years demonstrated a better functioning status and had more favorable HRQoL physical health over time. On the other hand, Jansson et al. [11] found that the age- and sex-matched sample population did not reach the required significance level which contradicted previous reports. Furthermore, investigations are warranted to determine the true effect of age on HRQoL after surgical intervention.

A study conducted among American patients who underwent arthroscopic rotator cuff repair found that the female gender was the only significant predictor of poor mental health HRQoL postoperatively while diabetes and poor engagement in sports activity contributed as well to the trend of worsening mental health [9]. This is almost consistent with our results, as we found that females demonstrated worse emotional well-being compared to males after the surgery, but physical health domains provided promising results, although the overall results did not reach statistical significance (p>0.05).

Moreover, patients with associated chronic diseases might be at the receiving end of a worst-case scenario postoperatively. Data in our study suggest that patients with underlying diseases exhibited poor overall physical component scores including its subdomains such as role limitations and pain. Similarly, this group of patients demonstrated weak social functioning but a higher score in fatigue. In Iran [13], a higher number of comorbidities, body mass index, adherence to physiotherapy, and postoperative complications were associated with HRQoL after total hip arthroplasty (THA), while in Brazil [17], using EQ-5D-3L showed significant improvement in QoL in the five dimensions of the instrument including mobility, self-care usual activities, pain/discomfort, and anxiety/depression. However, in Greece [14], body mass index, residence, social support, and education were not revealed to be relevant factors of QoL after THA. Chronic diseases could be a detrimental factor in patients after surgical interventions as they are prone to surgical complications and disease progression. Thus, healthcare providers had a significant role to monitor their rehabilitations and their adherence to follow-ups to alleviate any of the worst-case scenarios including the burden of the disease.

A survey carried out among Greek patients who underwent orthopedic surgery [11] revealed that for the overall mean EQ-5D score at baseline, procedures for benign or malignant tumors and elbow/hand diseases had statistically significantly higher scores than average while procedures related to hip and spine scored significantly less than the average. After 12 months of follow-up, the EQ-5D index score revealed an increase significantly by at least 0.18 units from baseline to 0.72 after 12 months. They further indicated that the mean EQ-5D in women also increased almost in parallel to that of men. In our study, although patients who underwent ankle surgery showed improved physical functioning and patients who underwent arm surgery exhibited better general health, patients who underwent hand surgery demonstrated significantly weak physical functioning as well as poor general health. Similarly, patients who underwent ankle surgery demonstrated fewer limitations due to emotional problems but not patients who underwent arthroplasty. No other QoL domains showed significant association with the type of surgery being performed on patients and therefore, it warranted further investigations. In Australia [16], patients with prosthetic joint infection (PJI) treated with debridement and prosthesis retention yielded good cure rates with PJI was revealed as a non-significant risk factor for poor quality of life which was based on univariate and multivariate regression estimates.

The study has some limitations including (1) the very small proportion of female patients in the sample, therefore, lacking the proper representation of female patients, and (2) no inclusion of other socio-demographic information such as BMI, marital status, employment status, smoking history, and annual income, etc., which could influence the quality of life. Therefore, we recommend that these limitations should be addressed in future prospective studies.

## Conclusions

The quality of life of patients after orthopedic surgical procedures seems to show improvements in physical health rather than mental health. Patients with associated chronic diseases tend to exhibit a lower quality of life postoperatively as compared to other patients; patients who underwent hand surgery demonstrated the worst-case scenario in terms of physical functioning and general health. Nevertheless, it is assumed that patients who underwent ankle surgery may have better HRQOL after the surgery both physically and emotionally. The quality of life after the surgery is an important indicator of patient satisfaction which may have a direct impact on the future outlook of a patient. More research is needed to determine the overall quality of life in patients who underwent a surgical procedure in our region.
